# Fecal Metagenomes and Metagenome-Assembled Genomes from the South American Sea Lion (Otaria flavescens) from the Comau Fjord (42°S), Patagonia

**DOI:** 10.1128/mra.00183-23

**Published:** 2023-04-17

**Authors:** Sergio Guajardo-Leiva, Valentín Berríos-Farías, Felipe G. Bermúdez, Eduardo Castro-Nallar

**Affiliations:** a Departamento de Microbiología, Facultad de Ciencias de la Salud, Universidad de Talca, Talca, Chile; b Centro de Ecología Integrativa, Universidad de Talca, Talca, Chile; c Center for Bioinformatics and Integrative Biology, Universidad Andrés Bello, Santiago, Chile; Montana State University

## Abstract

Environmental disturbances can be monitored using sentinel species. We present 30 temporally explicit metagenomes and 166 metagenome-assembled genomes (MAGs) from the gut of the South American sea lion (Otaria flavescens) to further understanding of whether variations in the gut microbiome composition and gene content might reflect environmental disturbances from salmon farming.

## ANNOUNCEMENT

Sentinel species are those that are sensitive to environmental disturbances and can be used in biological assessments (1). The Comau Fjord is surrounded by national parks and privately protected land that, however, is dotted with open-cage aquaculture centers that pour antibiotics into the ecosystem (2, 3). The South American sea lion (Otaria flavescens) is an opportunistic carnivore species that feeds on fish, crustaceans, and cephalopods and can be used as a sentinel of resource distribution (4–6). However, its use as a sentinel of anthropogenic impact has not been evaluated.

We collected fresh fecal samples off the ground (rocky outcrop) from a sea lion colony located in the Comau Fjord (42°S) for over 1 year. Samples were kept frozen until processing, which took place under a biohood (biosafety level 2 [BSL2]); a 250-mg aliquot was taken with a sterile spatula for DNA extraction using the Qiagen PowerFecal DNA extraction kit. Sequencing libraries were constructed with an Illumina TruSeq DNA kit and were sequenced with an Illumina NovaSeq 6000 system (150-bp paired-end reads) (https://doi.org/10.6084/m9.figshare.22233313.v4).

We used default software parameters unless otherwise stated. We removed adapters (–detect_adapter_for_pe) and carried out filtering and trimming (–q 30 –l 100) using fastp v0.21.0 (7). To remove host sequences, we filtered out all of the reads that mapped against some of the genomes most closely related phylogenetically to Otaria flavescens in the Otariidae clade that are available in the NCBI databases (Arctocephalus gazella, Callorhinus ursinus, Eumetopias jubatus, and Zalophus californianus) by using Bowtie2 (–end-to-end –un-conc) (8). We first estimated Mash distances using Mash v2.3 and checked whether metagenomes formed clusters using the Hopkins statistics (>0.75) (9). We then used agglomerative hierarchical clustering (hclust function in the base R package), which resulted in two clusters, as suggested by silhouette analysis. *De novo* metagenome coassembly was carried out on each of the two clusters, as implemented in MEGAHIT v1.2.9 (–min-contig-len 500) (10, 11). To bin the resulting contigs (829,689 contigs), we used VAMB v3.0.2, CONCOCT v1.1.0, and MetaBAT2 v2.15 separately to then refine the bins using the binning refinement module implemented in MetaWRAP v1.3.2 (–c 50 –x 10) (12–15). The full set of 166 metagenome-assembled genomes (MAGs) were quality assessed by running CheckM v1.2.0, which indicated 98 high-quality MAGs and 68 medium-quality MAGs (16).

Taxonomic assignment was conducted with GTDB-tk v1.5.1 and the database vR207 (17). To estimate the MAG abundances among the 30 sea lion gut metagenomes, we estimated the contig mean coverage per MAG by using CoverM v0.6.1 with the trimmed means method (https://github.com/wwood/CoverM). MAGs were previously dereplicated with an average nucleotide identity (ANI) threshold of 99% by using the Galah method and choosing the representative genome on the basis of CheckM genome quality values (18) (Table 1).

**TABLE 1:** Summary statistics for metagenome-assembled genomes

Parameter	Data for cluster:	
	C1	C2
No. of samples	21	9
No. of contigs	375,035	454,654
Total length (bp)	411,083,245	658,207,179
Minimum length (bp)	500	500
Maximum length (bp)	1,173,029	336,445
Mean length (bp)	1,096.1	1,447.7
No. of refined MAGs	85	81
No. of high-quality MAGs	54	44
No. of medium-quality MAGs	31	37

At the phylum level, the most abundant MAGs belonged to *Firmicutes* A (35.63%), *Fusobacteriota* (20.42%), *Firmicutes* (13.81%), *Proteobacteria* (13.55%), *Bacteroidota* (6.41%), *Campylobacterota* (6.12%), and *Actinobacteriota* (2.36%). At the family level the most abundant MAGs belonged to *Fusobacteriaceae* (20.44%), *Lachnospiraceae* (12.20%), *Clostridiaceae* (9.81%), *Moraxellaceae* (7.62%), *Bacteroidaceae* (5.61%), *Planococcaceae* (5.30%), and *Mycoplasmoidaceae* (5.23%).

### Data availability.

This whole metagenome shotgun project has been deposited in GenBank under the accession number PRJNA735411. The version described in this paper is the first version. Accession numbers for MAGs are provided in the supplemental table at https://doi.org/10.6084/m9.figshare.22233313.v4.
